# Cerebellar Medulloblastoma in Middle-to-Late Adulthood

**DOI:** 10.1155/2018/5425398

**Published:** 2018-01-31

**Authors:** Majid Aljoghaiman, Mahmoud S. Taha, Marwah M. Abdulkader

**Affiliations:** ^1^Department of Neurosurgery, King Faisal University, Al Ahsa, Saudi Arabia; ^2^Department of Neuroscience, King Fahad Specialist Hospital, Dammam, Saudi Arabia; ^3^Department of Pathology and Laboratory Medicine, King Fahad Specialist Hospital, Dammam, Saudi Arabia

## Abstract

Medulloblastoma is a malignant brain tumor that is typically seen in children. It is classified as an embryonal tumor, classically located within the posterior fossa. When it involves the fourth ventricle, the patient commonly presents with signs and symptoms of raised intracranial pressure secondary to obstructive hydrocephalus. It is exceedingly rare for Medulloblastoma to occur in middle and late adulthood. In this paper, we present a case of a 51-year-old man who presented with a posterior fossa mass that was diagnosed later as Medulloblastoma.

## 1. Introduction

Medulloblastoma is a malignant brain tumor (WHO grade IV) that is thought to arise from stem cells located in the subependymal matrix or the external granular layer (EGL) of the cerebellum [1]. It typically affects children and constitutes more than 25% of all pediatrics brain tumors [2]. There are some reported adult cases in the literature, accounting for less than 1% of brain tumors above the age of 18 [3]. The average incidence of Medulloblastoma is estimated at 1.5 per million population in the United States. Children of 1–9 years of age had an incidence rate of 6 compared to 0.6 in adults [4]. Medulloblastoma cases arise frequently in the midline, especially in the posterior vermis, adjacent to the roof of the fourth ventricle. Interestingly, the majority of adult cases are hemispheric in origin [5]. Clinically, these tumors fill the 4th ventricle and present insidiously with signs and symptoms of elevated intracranial pressure secondary to obstructive hydrocephalus.

## 2. Case Report

### 2.1. Clinical History

A 51-year-old male, known case of DM type II on insulin therapy, presented with history of repeated vomiting associated with progressive gait imbalance over one-month period.

### 2.2. Physical Examination

Neurological examination was normal apart from positive Romberg sign and ataxic gait. General physical examination and review of systems were essentially unremarkable.

### 2.3. Investigations

The initial laboratory and radiological work-up were not helpful. Brain CT scan at the emergency department showed abnormality of the 4th ventricle with a suspicious right middle cerebellar peduncle mass that warranted further assessment by MRI. An additional metastatic work-up to find a primary malignancy source was also negative.

### 2.4. Imaging

**Figure 1:**
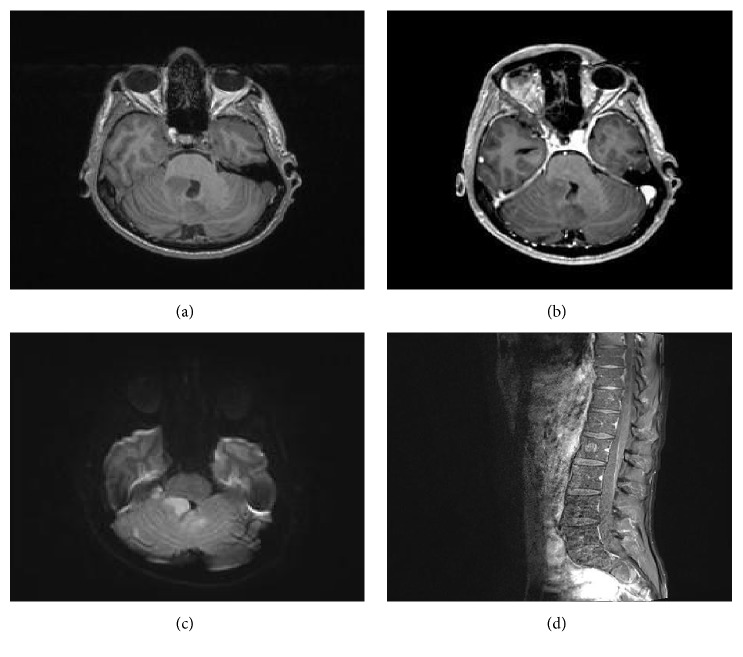
(a) Brain MRI T1 before contrast. (b) Brain MRI T1 after contrast. (c) FLAIR. (d) Spine MRI T1 after contrast.

Brain MRI before and after contrast (Figures 1(a), 1(b), and 1(c)) revealed an ill-defined lesion at the superomedial aspect of the right middle cerebellar peduncle distorting the upper aspect of 4th ventricle and the adjacent folia pattern. Another smaller lesion was also seen at the superior aspect of the vermis with perilesional high T2 and FLAIR signal. Both lesions showed an intermediate T2 and FLAIR signal intensity and faint, heterogeneous post-IV contrast enhancement, and relative restricted diffusion. Whole spine MRI (Figure 1(d)) showed interrupted leptomeningeal contrast enhancement anterior to spinal cord with focal high T2 signal intensity. The overall findings were highly suggestive of drop metastasis (sugar coating).

### 2.5. Pathology

**Figure 2:**
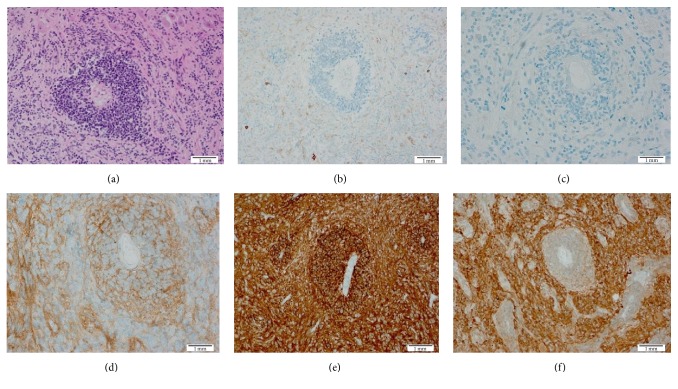
(a) H&E stain illustrating the primitive cells and the peculiar angiocentric pattern of distribution. (b) Immunohistochemical stain with CD45 (LCA) showed absence of immunoreactivity excluding lymphoma. (c) Pancytokeratin immunohistochemical stain was nonimmunoreactive excluding a metastatic carcinoma. ((d) and (e)) CD56 and CD99 immunohistochemical stains showed strong membranous pattern of immunoreactivity of the tumor cells. (f) Synaptophysin showed weak immunoreactivity.

The histological features seen in our case did not show the classical architectural pattern of Medulloblastoma. On the contrary, there were angiocentric arrangement and peculiar perivascular accentuation (Figure 2(a)) commonly seen in CNS lymphoma and other glial tumors such as ependymomas. The biopsy was composed of small round blue cells with hyperchromatic nuclei and indistinct cell borders. Many tumor cells were elongated and exhibited carrot-shaped cell morphology. Classical Homer-Wright rosettes were not present. The background cerebellar cortex was infiltrated by tumor cells, which also showed subpial accumulation and extension to the subarachnoid spaces. There was no evidence of large cell changes, significant cytological anaplasia, or desmoplasia.

Immunohistochemical studies showed focal GFAP positivity, diffuse weak positive staining for synaptophysin, and strong positivity for INI-1 and CD56. NSE and neurofilament protein immunohistochemical markers showed strong cytoplasmic staining in the majority of the tumor cells. Retinal S antigen was not available in our laboratory to perform. We did other immunohistochemical stains such as LCA (CD45) which was negative excluding the diagnosis of lymphoma. Pancytokeratin stain was nonimmunoreactive excluding a metastatic carcinoma. Immunohistochemical surrogate markers such as GAB1, YAP1, *β*-catenin, and Filamin A are reliable tools in subclassifying Medulloblastoma. *β*-catenin (nuclear pattern), YAP1, and Filamin A immunoreactivity characterize the WNT subtype, while GAB1 immunoreactivity indicates an SHH subtype. The non-SHH/non-WNT subgroup only shows cytoplasmic *β*-catenin staining while the remaining markers are nonimmunoreactive. In our case the tumor showed cytoplasmic *β*-catenin without nuclear immunoreactivity and GAB1 positivity. It was negative for YAP1. These results were compatible with a SHH molecular subtype. P53 immunohistochemical antibodies were our method to assess P53 mutation; the neoplastic cells were nonimmunoreactive, indicating that this tumor is TP53 wild type.

### 2.6. Follow-Up/Outcome

The clinical stage of the disease and the status of the patient precluded any further surgical intervention. The patient then received a combination of chemotherapy and radiotherapy. Vincristine was the choice of chemotherapy, while radiotherapy with 15 fractions and total dose of 27 Gy was also given. The patient, however, continued to deteriorate and eventually passed away 2 months later.

## 3. Discussion

Medulloblastoma (WHO grade IV) is the second most common brain tumor in children after pilocytic astrocytoma [1]. Although the exact etiology and pathogenesis of this tumor are not entirely understood at present, some recent studies have provided insights into possible disease mechanisms. Some suggested that the tumor developed from the remnants of the embryonic cells found in the external granular layer of the cerebellum [5]. Medulloblastoma has two peaks of incidence, the first in the first decade of life and the second in the early twenties. It represents less than 1% of all CNS tumors in the adult population. Radiologically, the MRI imaging of Medulloblastoma usually shows a compact, isointense mass, occasionally with a cystic component. Its heterogeneous contrast enhancement reflects the rich vascularity and the dense cellularity of these tumors. Our case's radiological features were rather atypical and subtle; the mass was located in the right middle cerebellar peduncle involving the superior vermis and indenting rather than filling the fourth ventricle.

Currently, the WHO tumor classification recognizes four distinct histological subtypes (classical, desmoplastic/nodular, Medulloblastoma with extensive nodularity, and large cell anaplastic Medulloblastoma). Furthermore, the new WHO tumor classification 2016 has classified Medulloblastoma according to its molecular properties. Four subgroups are now identified, the Wingless (WNT), Sonic Hedgehog (SHH), group 3, and group 4 [6]. The desmoplastic/nodular histological pattern is seen almost exclusively in the SHH subgroup. This molecular subgroup has a good-to-intermediate prognosis. The WNT subgroup has the best overall survival rate followed by SHH and group 4 whereas group 3 has the worst prognosis and carries the highest rate of metastasis at the time of diagnosis. It is noted that most of the adult reported cases are of the classical histological type. Similarly, our case showed a classical histological type and was subclassified in the SHH molecular subgroup. Only two cases reported by Ramsy et al. were of desmoplastic variant [7, 8].

Several case reports have been published of patients diagnosed with Medulloblastoma in middle-to-late adulthood. Cervoni and colleagues reported two cases of Medulloblastoma aged 71 and 67 with one of them surviving up to 42 months after surgical resection and radiotherapy with no evidence of recurrence [9]. The oldest patient reported in the literature with Medulloblastoma was reported by Kepes et al. at age of 73 [10]. More cases of Medulloblastoma above the age of 50 are summarized in Table 1. Most of the cases reported in the elderly (including ours) occurred in the hemisphere of the cerebellum, presumably due to migration of the undifferentiated neuroblasts from the posterior medullary velum laterally [5]. Only 2 cases were reported in the vermis and in the 4th ventricle by Jaiswal et al., respectively [11, 12].

Dissemination of Medulloblastoma within CSF pathways is a defining pathobiologic characteristic of this tumor; when present it signifies a decimal prognosis. Treatment options include complete total surgical resection plus craniospinal irradiation. Chemotherapy is another treatment modality, though its effectiveness in the management of Medulloblastoma is still controversial. Subtotal surgical resection and evidence of disease dissemination are considered poor prognostic factors [13]. Five-year survival for Medulloblastoma in adults has been estimated to be 46% to 78% as per reports by Skolyszewski and Glinski [14]. In our case, after the diagnosis was established, the patient was immediately treated with combined radiotherapy and chemotherapy. Unfortunately, the patient did not recover and continued to deteriorate until he passed away 2 months after the surgery.

In conclusion, although Medulloblastoma is a pediatric tumor and far less common than metastasis or lymphoma in a posterior fossa mass in an adult, it should be considered in the differential diagnoses.

## Figures and Tables

**Table 1:** Cases of Medulloblastoma in patients above the age of 50.

Study	Author	Age	Location	Pathology
(1)	Cervoni	71	R cerebellar hemisphere	Not specified
(2)	Cervoni	67	L cerebellar hemisphere	Not specified
(3)	Jaiswal	65	Vermis	Medulloblastoma with glial differentiation
(4)	Huppmann	65	R cerebellar hemisphere	Classic subtype
(5)	Yong	71	4th ventricle	Classic subtype
(6)	Kepes	73	R cerebellar hemisphere	Classic subtype
(7)	Ramsay	66	Cerebellar hemisphere	Classic subtype
(8)	Ramsay	65	Cerebellar hemisphere	Desmoplastic subtype
(9)	Liang	72	R cerebellar hemisphere	Classic subtype
(10)	Sajko	62	R superior cerebellar peduncle	Desmoplastic subtype
(11)	Aljoghaiman (current case)	51	R middle cerebellar peduncle + superior vermis	Classic subtype (SHH subgroup)
